# Effect of Photobiomodulation in Patients with Temporomandibular Dysfunction Refractory to Botulinum Toxin Treatment: A Non-Controlled Multicentric Pilot Study

**DOI:** 10.3390/jcm14113778

**Published:** 2025-05-28

**Authors:** José Antonio Blanco Rueda, Antonio López-Valverde, Antonio Marquez-Vera, Natàlia dos Reis Ferreira, Bruno Macedo de Sousa, Nansi López-Valverde

**Affiliations:** 1 Department of Surgery, University of Salamanca, 37008 Salamanca, Spain; jablancor@usal.es (J.A.B.R.); nlovalher@usal.es (N.L.-V.); 2 Biomedical Research Institute of Salamanca (IBSAL), 37007 Salamanca, Spain; 3 Department of Nursing & Physiotherapy, University of Salamanca, 37008 Salamanca, Spain; amarvefisio@usal.es; 4 Institute for Occlusion and Orofacial Pain, Faculty of Medicine, University of Coimbra, 3045-093 Coimbra, Portugal; natalia.ferreira@fmed.uc.pt (N.d.R.F.); bsousa@fmed.uc.pt (B.M.d.S.)

**Keywords:** temporomandibular joint disorders, low-level laser (light) therapy, photobiomodulation, botulinum toxin, pilot project

## Abstract

**Background/Objectives**: Temporomandibular disorders are a heterogeneous group of degenerative musculoskeletal conditions that present a series of symptoms such as pain, dysfunction of the masticatory muscles and/or temporomandibular joints, structural abnormalities, and limitation or alteration of mandibular movements. The objective of this study was to evaluate the efficacy of photobiomodulation therapy with low-power laser in patients refractory to treatment with botulinum toxin type A. **Methods**: A multicenter prospective experimental pilot study was proposed, in which 10 patients between 33 and 68 (50 ± 11.2) years old participated, assigned to a laser group (940 nm diode laser) who had previously been treated with a minimum of three doses of botulinum toxin type A without obtaining positive results. The patients underwent four photobiomodulation sessions over 4 weeks (registered at ClinicalTrials NCT06915064). Painful symptoms were evaluated using a visual analog scale at different locations, the pressure pain threshold using algometry, and the maximum vertical mandibular movement determined using digital calipers. The results were recorded four weeks after the end of treatment. Adverse effects were also evaluated. **Results**: Pain in the masticatory muscles was reduced in 70% of patients with statistically significant values (*p* = 0.002); a total of 60% of patients showed a considerable reduction in joint noise with outstanding statistical significance (*p* = 0.015). The majority of participants reported a reduction in the intensity of headaches after treatment. However, it only produced a slight improvement in maximum mouth opening and lateral excursions. Algometric values in the masticatory muscles showed improvement only in the left-sided irradiated muscles. **Conclusions**: Photobiomodulation therapy is a non-invasive treatment option for temporomandibular disorders that generates positive effects in cases refractory to treatment with botulinum toxin type A.

## 1. Introduction

Temporomandibular disorders (TMDs) are a heterogeneous group of degenerative musculoskeletal conditions, affecting a significant proportion of the population and presenting with a range of symptoms such as pain, dysfunction of the masticatory muscles and/or temporomandibular joints (TMJs), structural abnormalities, and limitation or alteration of jaw movements [1].

Their clinical management has been addressed by non-invasive, minimally invasive, and fully invasive treatments. The most common noninvasive treatments are physical therapy, occlusal splints and/or occlusal adjustments, and drugs. Minimally invasive treatments include sodium hyaluronate and corticosteroid injections, arthrocentesis, and arthroscopy. Finally, in patients in whom nonsurgical treatments fail, open TMJ surgery may be necessary to restore function and relieve pain [2].

Botulinum toxin (BTX), with forty different serotypes, is one of the most potent toxins and is produced by Clostridium botulinum. In the treatment of TMDs, serotype A is the most commonly used, along with serotype B, which is used occasionally [3]. However, it is not considered a first-choice treatment, mainly due to its adverse effects, limited efficacy, and lack of objective evaluation [4].

Light amplification by stimulated emission of radiation (LASER), created in the 1960s by Theodore Maiman [5], was immediately recognized for its many potential applications in medicine, and its different varieties are currently used in a wide range of clinical applications. The low-level laser (LLL) produces monochromatic and coherent light of a single wavelength, with anti-inflammatory action through a photochemical effect (photobiomodulation therapy, PBMT) [6]. Laser therapy can promote faster tissue healing by inducing neovascularization, as well as reducing inflammation and pain, thanks to the photobiomodulatory effect it can have on cells and tissues. LLL emits a very small amount of energy and has become a technique of interest to dental professionals due to the aforementioned effects and its ability to reduce muscle spasms [7]. The use of LLL with wavelengths between 780 nm and 980 nm is considered safe and effective and seems to trigger additional photochemical reactions at the mitochondrial level, leading to a change in cellular metabolism and altered protein synthesis; this technique also shows effective tissue penetration and good relief of chronic pain [8]. However, some studies have also highlighted the benefits of PBMT with the simultaneous use of red and infrared LEDs on pain and jaw range of motion in people with TMDs [9].

The objective of our preliminary research was to identify the effect of PBMT-LLL in patients with myofascial TMDs refractory to BTX treatment. Specifically, we proposed the hypothesis of PBMT-LLL as a safe, low-cost, painless, and minimally invasive alternative therapy to improve complete muscle recovery and significantly reduce muscle paralysis in patients with masticatory muscle disorders, previously treated with BTX. In addition, we investigated the different treatment alternatives and suggested a protocol for the application of PBMT-LLL in myofascial TMDs and the pain it causes in the masticatory muscles, also analyzing its complications and negative effects.

## 2. Materials and Methods

### 2.1. Study Design and Registration

To address the research objective, the authors designed and carried out a non-controlled multicenter pilot study at the universities of Coimbra (Portugal) and Salamanca (Spain), in accordance with the Declaration of Helsinki, which was approved by the Drug Research Ethics Committee of the Salamanca Health Area (reference CEIm: 2023 07 1379, dated 22 January 2024). All patients received the corresponding informed consent form and authorized their inclusion in this study which is registered in Clinicaltrials NCT06915064. 

### 2.2. Patients: Inclusion and Exclusion Criteria

Ten female patients diagnosed with TMDs who attended the Department of Maxillofacial Surgery at the University Hospital Complex of Salamanca were included in this study. All presented with uni- or bilateral painful symptoms associated with bruxism, hypertrophy of the masticatory muscles and headaches, and had previously been treated with BTX (minimum 3 doses of 100 IU in masticatory muscles and in the intracapsular area), without obtaining favorable results. Patients who had not previously been treated with this type of treatment, those who were being treated in the Pain Unit, and patients undergoing invasive treatments, such as open surgery in the cervicofacial region or arthrocentesis of the TMJs, were excluded. During treatment, patients did not undergo restorative or prosthetic dental treatment, nor did they use orthodontic or functional orthopedic appliances, myofunctional plates or insoles, or any analgesic or anti-inflammatory drugs.

### 2.3. Assessment Tools

Psychosocial aspects such as stress or anxiety can increase muscle tension in the jaw, which can trigger disorders of the TMJs, myofascial pain, and bruxism, so the DASS-21 (Depression Anxiety Stress Scales-21) questionnaire [10] was used to assess affective states through the 21 questions that make up the tool. To evaluate both the severity and the frequency of the symptoms associated with TMJ disorders, the anamnestic index of Fonseca was used, which is considered a valid tool for diagnostic confirmation [11]. For the evaluation of pain, the visual analog scale (VAS) was used, which provides a subjective evaluation of pain, with a scale of values from 0 to 10 representing the intensity of pain in an ascending manner [12]. To measure the maximum mouth opening (MMO), a digital gauge (Ubermann^®^, Urubamba, Peru) was used, taking two measurements, and the average was used for statistical analysis. Pressure pain (PP) was measured using a pressure algometer (Wagner FPI 10^®^, Greenwich, CT, USA) that shows its strength during manual therapy on pressure points (tip of the algometer perpendicular to the muscle, maintaining the pressure which is progressively increased up to 1 kg/s). Joint noises and creaking were assessed subjectively by the clinical evaluator (TMJ auscultation) and objectively by the patient, without measuring the intensity of the symptom. At the end of this study, the degree of satisfaction with the treatment received was evaluated in the patients. For this purpose, the Likert scale was used [13], which is a validated scale consisting of 5 items that evaluate attitudes, perceptions, or levels of satisfaction.

### 2.4. Treatments

**Figure 1:**
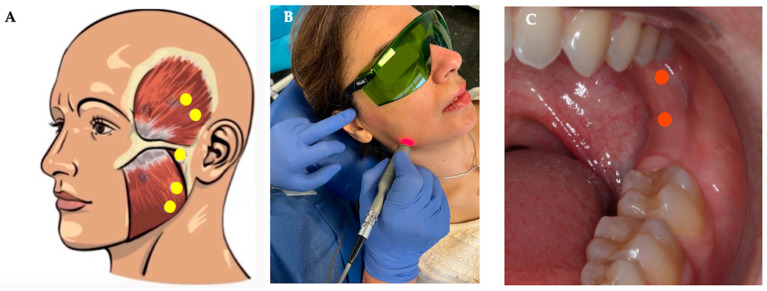
(**A**) drawing of the extraoral application areas of EPIC X laser radiation; (**B**,**C**) photographs of extraoral and intraoral application, respectively.

Three patients (30%) received treatment on the left side and seven (70%) on the right side, as these were the sides where they felt pain. The treatment was carried out using the EPIC X laser (BIOLASE^®^, Foothill Ranch, CA, USA), which uses a solid-state diode as a semiconductor source of invisible infrared radiation (Table 1). The procedure for the treatment of myofascial pain was carried out by topical heating, to raise tissue temperature, for temporary relief of pain and muscle and joint stiffness, as well as to produce an increase in local circulation and temporary muscle relaxation. The therapy was applied to the masticatory muscles: temporalis (medial and posterior bundle), masseter (lateral and medial area of the mandibular angle), internal pterygoid (behind the retromolar trigone towards the medial aspect of the mandible), and in the TMJ area (posterior area in front of the external auditory canal) to directly activate the painful points discovered on palpation (Figure 1). All patients included in this study underwent four sessions of PBMT-LLL for four consecutive weeks. The final evaluation was carried out one month after the last PBMT-LLL session (Figure 1).

### 2.5. Data Collection and Statistical Analysis

Data collection and analysis were carried out by an independent examiner. The data were compiled and tabulated in an Excel spreadsheet. Descriptive statistics were performed (IBM SPSS Statistics 26, 2019, Inc., Chicago, IL, USA), considering the treatment and the patient as a unit of analysis. Frequency distributions were calculated for qualitative variables and mean values and standard deviations for quantitative variables. Binary variables were used to analyze the clicks and joint noise parameters. The Student’s *t*-test and the normality test were selected to compare the values of the parameters considered, before and after the application of PBMT-LLL, with a statistical significance threshold of <0.05.

## 3. Results

There were no dropouts, so the sample consisted of 10 female patients with a mean age of 50 ± 11.2 years. All patients included in this study presented, prior to treatment, with symptoms compatible with TMD. Nine patients (90%) presented joint creaking, four (40%) headaches, and eight (80%) pain. Only one of the patients included in this study (10%), according to the DSS-21 questionnaire, reported moderate anxiety (Table 2).

**Figure 2:**
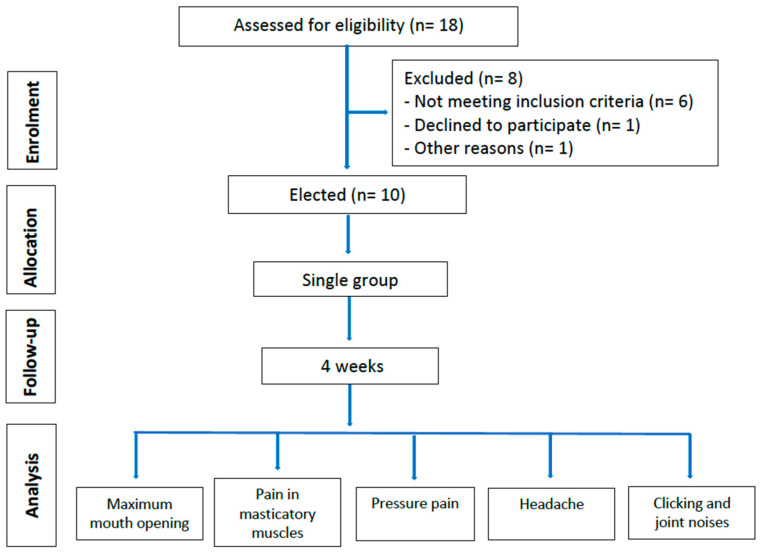
Flow chart of progress through the phases of this study according to the CONSORT statement.

The study flow diagram was prepared according to the CONSORT guidelines [14] and is shown in Figure 2.

### 3.1. MMO

**Figure 3:**
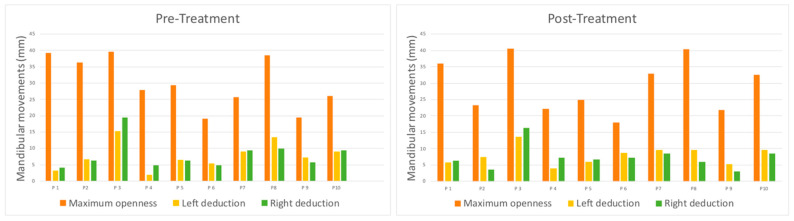
MMO pre-treatment and post-treatment graphs.

Before treatment with PBMT-LLL, all the patients included in the sample showed a significant reduction in the mean parameters of MMO (40–45 mm) and lateral deviations (10–12 mm). Treatment with PBMT-LLL only produced a slight improvement in MMO in 50% of the patients and in 40% of the left and right deviations. The t-test for paired samples did not find statistical significance in any of the cases (*p* = 0.657 maximum openness; *p* = 0.763 left diduction; and *p* = 0.422 right diduction) (Figure 3, Table 3).

### 3.2. Pain in Masticatory Muscles

**Figure 4:**
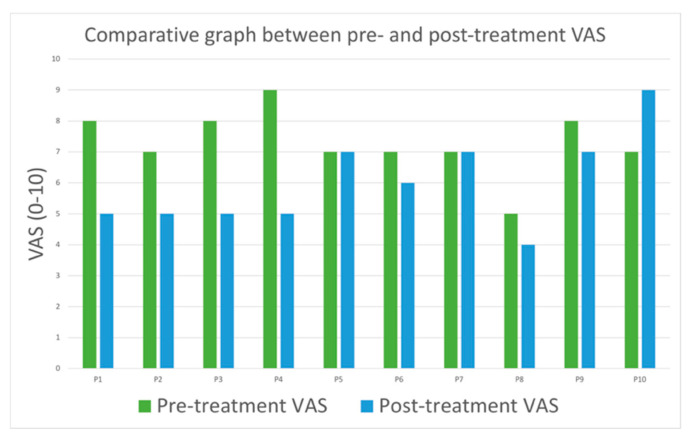
Graph demonstrating the evolution of pain in the masticatory muscles before and after treatment.

Before treatment, pain assessment using VAS showed average values of 4.6 in the temporalis muscle, 4.3 in the masseter, 6.7 in the internal pterygoid, and 4.2 in the intra-articular area of the TMJs. After treatment with PBMT-LLL, pain in the masticatory muscles was reduced in 70% of patients. Only one (10%) showed increased pain in the masticatory muscles after treatment (statistical significance *p* = 0.002) (Figure 4, Table 4).

### 3.3. PP

**Figure 5:**
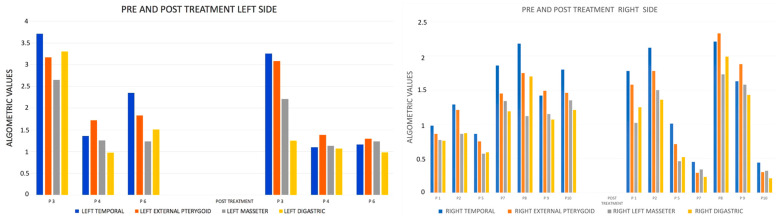
Graph of algometric VAS values in the masticatory muscles before and after treatment.

The algometric VAS values with PBMT-LLL treatment of the masticatory muscles (temporal, masseter, external pterygoid, and digastric) showed an improvement in the irradiated muscles on the left side; however, on the right side there was no such improvement, and, in general, except in two patients (20%), the results were unfavorable (Figure 5, Table 5 and Table 6).

### 3.4. Headache

**Figure 6:**
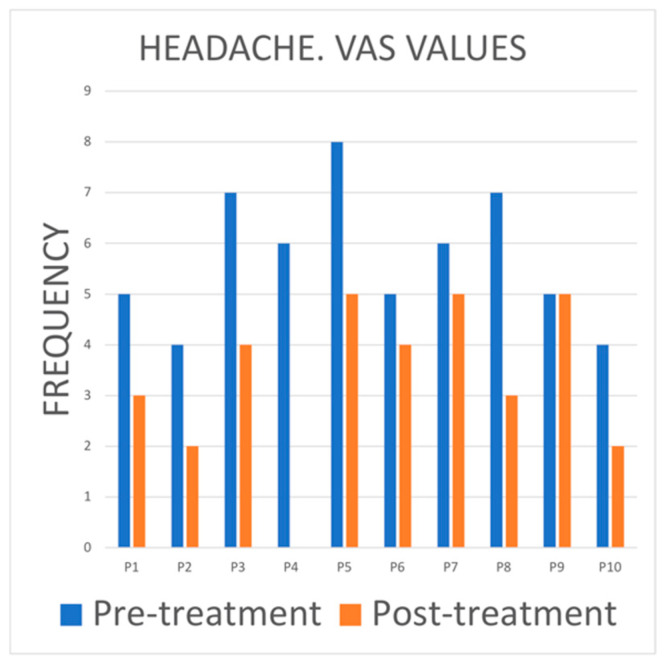
Bar chart showing VAS scores for headache pain. Before and after treatment.

Most participants reported a reduction in headache intensity after treatment. In some cases, the reduction was more noticeable (P5 and P8), indicating that PBMT-LLL treatment could be effective in reducing headache in participants (Figure 6).

### 3.5. Clicks and Joint Noises

**Figure 7:**
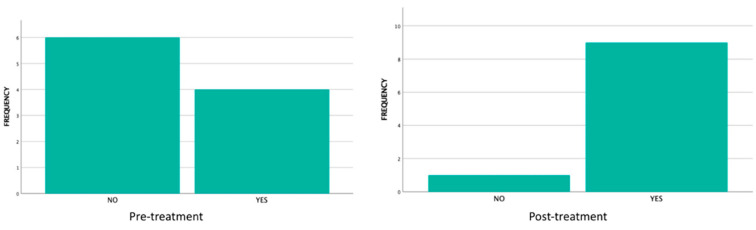
Evolution of clicks and joint noises before and after treatment.

At the start of this study, nine patients (90% of the sample) presented clicks/joint noises. After treatment, 60% of patients reported an improvement (statistical significance *p* = 0.015) (Figure 7, Table 7).

### 3.6. Likert Scale

Four patients expressed their total satisfaction and three agreed with the care and treatment they received; one disagreed, one was indifferent, and one did not answer (Table 8).

### 3.7. Adverse Events

In this pilot study, patients did not report any adverse effects to the therapy received. Only one patient (10%) complained of mild itching in the treated area the day after treatment.

## 4. Discussion

A randomized controlled trial (RCT) is considered the best study to demonstrate cause–effect in any clinical area; however, its design and implementation is a complex task that involves, in addition to time, economic costs. For this reason, it is advisable to carry out a pilot study before the main study to increase the chances of success and save time and economic costs [15]. In itself, the pilot study is a small-scale investigation, with a reduced sample size, aimed at testing procedures and methods that are useful for subsequent use in a large RCT. Pilot trials focus on assessing feasibility rather than effectiveness or efficacy. All of this justifies a pilot study preliminary to an RCT [16].

In a previous study [17], we presented the results of the effects of BTX in intramuscular and intra-articular injections, but despite the results obtained, a percentage of patients, approximately 25 to 30%, were refractory to treatment or rejected the option of invasive treatment. Furthermore, despite the existence of multiple procedures to treat the symptoms of TMDs, no specific treatment has consistently demonstrated its effectiveness. However, a recent review by Popescu et al. [18] highlights the need to use ultrasound to improve the accuracy of BTX injections, as the guided technique would allow for precise needle placement, minimize adverse effects related to product diffusion, and enable quantitative and serial assessment of muscle thickness and texture, thereby helping to differentiate refractory cases due to technical failures from those caused by the failure of the drug applied.

In the pilot study, we present that PBMT-LLL treatment reduced pain in the masticatory muscles and pressure pain in patients treated on the left side, as well as significantly reduced headaches and joint noises. However, it only produced a slight improvement in the MMO, both in the mouth opening and in the left and right diduction. These results are consistent with other previous studies that did not find great benefits from PBMT-LLL treatment of TMDs [19,20,21].

TMDs are one of the main causes of orofacial pain; furthermore, the importance of gender in TMD development has been demonstrated, with women being twice as likely to develop it as men, and in our study, the total sample was female, which could have influenced the results [22,23]. In our study, pain in the area of the masticatory muscles and in the intra-articular area of the temporomandibular joints (TMJs) was evaluated before and after treatment with PBMT-LLL, and a reduction in pain was observed in 70% of the patients included in this study, with statistically significant values (*p* = 0.002). These results coincide with those of other studies with large samples, irradiated areas, and follow-ups similar to ours [24,25], which is probably due to the biostimulant, analgesic, and muscle relaxant effects of radiation. However, there are discrepancies regarding the points of application and there seems to be support for the idea that direct application to the points of maximum pain, as was carried out in our study, would produce better results than traditional punctual radiation, due to its focused application [25].

PP is the most widely used method for detecting pressure sensitivity and static mechanical allodynia in deep muscles [26]. In our study, we did not find significant results and only more favorable results in patients who were treated on the left side (30%), although we do not know what could explain the difference in results with the right side. However, in line with our results, an RCT with a sample of 91 patients also found no differences in PP between the PBMT-LLL-treated groups and the placebo group [27]. Nor did we find statistically significant results on the effectiveness of PBMT-LLL on the MMO and the lateral excursions of the mandible, with only a slight improvement in the MMO in 50% of the patients treated and in 40% of the patients in terms of the left and right lateral movements. These results would disagree with other studies that reported significant improvements in functional aspects related to jaw mobility through the treatment of TMDs with LLL [28,29]. It is possible that these results were due to unilateral treatment; however, a recent meta-analysis of 24 RCTs reported the limited effect of laser therapy in improving jaw movement in patients with TMDs [30]. All these results demonstrate the controversies that exist regarding its effectiveness in certain situations.

Pain is usually the main reason why patients with TMDs seek treatment, and pain relief improves the restriction of jaw movements, together with the masticatory function of patients. Indeed, our study found that TMD therapy with PBMT-LLL significantly improved pain in the masticatory muscles (*p* = 0.002); this finding is consistent with the reports of most laser therapy studies on TMJ pain relief [21,27,30,31]. In this respect, it is worth mentioning that, in our study, as in other studies consistent with our results [32], the radiation was not only applied to the painful points located in the masticatory muscles but also to the TMJ area.

Comorbidities are often associated with TMDs [33] and, among the coexisting pathologies, headaches and migraines, among others, are often associated in a bidirectional way, since both pathologies increase the risk of suffering TMDs [34]. Some recent studies have even suggested that one condition could influence the development of another [35]. It has also been shown that the coexistence of both conditions shows higher levels of allodynia and hyperalgesia, possibly due to the sensitization of the central and peripheral nervous system and the deterioration of the pathways that modulate pain [36]. Our study revealed a significant reduction in the intensity of VAS scores for headaches after treatment in the majority of participants, which would indicate that PBMT-LLL treatment could be effective in reducing headaches in patients with TMD. Only one of the patients included in this study (10% of the sample) reported an increase in headaches after treatment, attributing it to the fact that, as the treatment was unilateral, the headache could have started on the untreated side.

Joint noises in the TMJs, with or without associated pain, are a really annoying symptom for patients who suffer from it, and, on occasion, it conditions their social relationships and quality of life [37]. In general, clinicians resort to mandibular exercise programs together with bite splints for treatment [38] and even stress reduction therapies since, in the etiology of TMDs, both the patient’s stress levels and those of anxiety and depression are triggers in situations that present with musculoskeletal pain [39]. In fact, the patients included in this pilot study were evaluated at the start of treatment using the DASS-21 tool [10], which showed mild anxiety levels in 10%. A recent Cochrane review of 3000 participants [40] reported uncertain evidence for the reduction in joint clicks in studies comparing occlusal splints and physiotherapy with LLL. However, we found that, at the start of this study, 90% of patients reported suffering from joint noises, and the evaluation four weeks after treatment showed that 60% of patients reported a significant reduction or disappearance, with outstanding statistical significance (*p* = 0.015). On the other hand, we have not found any study in the literature that directly relates LLL with the therapy of joint noises.

Our study had a number of limitations, which we will now describe: The small sample of patients. However, this was a pilot study and, according to the literature, the sample size was adequate. Our study did not include randomization, as we evaluated an intervention without randomizing the participants (feasibility study), which is something we will do in a future study with an adequate sample size to provide statistical power [41,42]. The follow-up of the patients was 4 weeks after the end of the treatment and, according to the Cochrane review [40], much longer-term follow-ups would be necessary, with a period of 5 years even being proposed. Patients only received treatment on one side (right or left), and outcomes were not compared. It is also worth noting that the included patients had received prior treatment with botulinum toxin (BTX) without obtaining the expected results. Finally, it should be noted that all the patients included were women, and their predisposition to orofacial pain is important to consider. All these limitations are being considered in an RCT currently being carried out in our Maxillofacial Surgery Department.

## 5. Conclusions

In summary, treatment of TMDs with PBMT-LLL in patients refractory to BTX treatment was promising in reducing pain in the masticatory muscles and joint noises, as well as headaches; however, these preliminary results need to be confirmed by RCTs. We are currently conducting further research with larger patient samples and long-term follow-ups to confirm the preliminary results of this pilot study.

## Figures and Tables

**Table 1:** Device and radiation.

Device	Year	Type	Wavelength (nm)	Out Power (W)	Tip Area	Laser Dose per Session at Each Point	Exposure Time per Point/ Session	Total Laser Dose per Session	Total Laser Dose per Treatment (4 Sessions)	Total Energy (10 Patients)
Diode laser EPIC X (BIOLASE^®^) Stellahelz, Biolase, Spain	2023	Epic X	940 nm	0.9 W	1.5 cm^2^ beam +/− tip	10.0 J/cm^2^	17 s.	105 J	420 J	4200 J

W, Watt (unit of power); J, fluence to energy per unit surface area.

**Table 2:** Baseline data.

Gender	Age	Employment Status	Side Treated	Symptoms of TMDs (Fonseca Index)	DASS-21	Joint Cracking	Headache	Pain
**♀** (100%)	50 ± 11.2 years	Working 80%	Right side 70%	100%	10% mild anxiety	90%	40%	80%

DASS-21, Depression Anxiety Stress Scales-21; TMDs, Temporomandibular disorders.

**Table 3:** *t*-test for MMO with paired samples (95% confidence interval).

		Mean	SD	*t*	*p*-Value
Pair 1	Pre-maximum openness–post-maximum openness	0.876	6.039	0.459	0.657
Pair 2	Pre-left diduction–post-left diduction	−0.219	2.226	−0.311	0.763
Pair 3	Pre-right diduction–post-right diduction	0.653	2.453	0.841	0.422

SD, standard deviation.

**Table 4:** *t*-test for pain in masticatory muscles with paired samples (95% confidence interval).

		Mean	SD	*t*	*p*-Value
Pair 1	Pre-treatment and post-treatment	2.400	1.713	4.431	0.002 *

SD, standard deviation; * statistical significance.

**Table 5:** *t*-test for algometric values (left side) with paired samples (95% confidence interval).

		Mean	SD	*t*	*p*-Value
Pair 1	Left temporal pre- and post-treatment	0.633	0.491	2.233	0.155
Pair 2	Left ext. pteryg. pre- and post-treatment	0.320	0.220	2.512	0.129
Pair 3	Left masseter pre- and post-treatment	0.190	0.226	1.456	0.238
Pair 4	Left digastric pre- and post-treatment	0.826	1.105	1.295	0.325

SD, standard deviation.

**Table 6:** *t*-test for algometric values (right side) with paired samples (95% confidence interval).

		Mean	SD	*t*	*p*-Value
Pair 1	Right temporal pre- and post-treatment	0.107	0.926	0.306	0.770
Pair 2	Right ext. pteryg. pre- and post-treatment	0.014	0.818	0.046	0.965
Pair 3	Right masseter pre- and post-treatment	0.030	0.718	0.110	0.916
Pair 4	Right digastric pre- and post-treatment	0.194	1.364	−1.411	0.208

SD, standard deviation.

**Table 7:** *t*-test for clicks and joint noises with paired samples (95% confidence interval).

		Mean	SD	*t*	*p*-Value
Pair 1	Pre-treatment and post-treatment	−0.500	0.527	−3.000	0.015 *

SD, standard deviation; * statistical significance.

**Table 8:** Evaluation of the degree of satisfaction according to the Likert scale.

Patients	Totally Agree	Agree	Neutral	Disagree	Strongly Disagree
1		x			
2		x			
3	x				
4	x				
5	x				
6			x		
7	x				
8				x	
9	-	-	-	-	-
10		x			

Questions: 1. Do you think the medical team understood and helped you understand your illness? 2. Are you satisfied with the medical team? 3. Do you think the medical team has understood your needs? 4. Are you generally satisfied with the treatments administered by the medical team? 5. In general, have you found relief from your illness?

## Data Availability

All data generated and analyzed, including the study protocol and search strategy, as well as included and excluded studies, extracted data, analysis protocols, and quality assessment, are available in the article and can be requested from the corresponding author.

## Abbreviations

TMD	Temporomandibular disorder
TMJs	Temporomandibular joints
BTX	Botulinum toxin
LLL	Low-level laser
PBMT	Photobiomodulation therapy
DASS-21	Depression Anxiety Stress Scales-21
VAS	Visual analog scale
PP	Pressure pain
MMO	Maximum mouth opening
RCT	Randomized controlled trial
